# Supervised Exposure to XR Device–Task Configurations in Mild Cognitive Impairment: Descriptive Self-Report Acceptability Pilot Study

**DOI:** 10.3390/s26154788

**Published:** 2026-07-28

**Authors:** Francesco Rundo, Sabrina Musso, Serafino Buono, Marilena Recupero, Raffaele Ferri

**Affiliations:** 1 Oasi Research Institute—IRCCS, Via Conte Ruggero n° 73, 94018 Troina, Italy; frundo@oasi.en.it (F.R.); smusso@oasi.en.it (S.M.); fbuono@oasi.en.it (S.B.); rferri@oasi.en.it (R.F.); 2 Department of Medicine and Surgery, “Kore” University of Enna, 94100 Enna, Italy

**Keywords:** Touch TV, CAVE, HMD, VR Headset, mild cognitive impairment

## Abstract

**Highlights:**

**What are the main findings?**

- Individuals with MCI and cognitively healthy controls reported generally favorable subjective acceptability for three XR device–task configurations: Touch TV, CAVE, and HMD.
- CAVE was numerically the most frequently selected configuration for future applications, especially in the MCI group, but this finding was descriptive and did not establish superiority over the other configurations.

**What are the implications of the main findings?**

- The findings support the self-report acceptability of exposing individuals with MCI to different XR-based IADL configurations under supervised conditions.
- Because device type, interaction modality, immersion level, and task content were not experimentally separable, these results should be interpreted only as preliminary acceptability data. Future studies require task–device counterbalancing, validated usability scales, cybersickness measures, and objective interaction metrics.

**Abstract:**

Extended reality (XR) platforms may support cognitive and functional interventions in mild cognitive impairment (MCI), but their clinical feasibility depends on perceived usability, comfort, and user acceptance. This exploratory pilot study examined subjective acceptability of three integrated XR device–task configurations: a non-immersive Touch TV configuration, a semi-immersive Cave Automatic Virtual Environment (CAVE) configuration, and a fully immersive head-mounted display (HMD) configuration. The study included 21 individuals with MCI and 10 cognitively healthy controls. Participants experienced all three device–task configurations in randomized order and completed the Digital Immersion Satisfaction Questionnaire (Qu.G.I.D.), an ad hoc instrument assessing digital literacy, perceived ease of use, preference, and willingness to use the configurations for future applications. Perceived ease of use scores were generally high and did not differ significantly between groups. Repeated-measures exploratory analyses showed no statistically robust configuration effect for perceived ease of use or preference scores. CAVE was numerically the most frequently selected device–task configuration for future applications, but this difference was not statistically significant when non-mutually exclusive selections were analyzed using Cochran’s Q. Digital literacy was not positively associated with Touch TV preference. Because device type, level of immersion, interaction modality, and task content were not experimentally separable, these findings cannot determine whether ratings reflected hardware characteristics or the specific activities performed. The results should therefore be interpreted as preliminary descriptive self-report acceptability and acceptability data, not as evidence of comparative device–task configurations effectiveness. Larger, balanced, counterbalanced studies using validated usability scales, objective performance metrics, interaction-quality measures, and cybersickness assessment are required before hypotheses for future controlled studies can be formulated.

## 1. Introduction

Mild Cognitive Impairment (MCI) represents a clinical condition of increasing epidemiological relevance, defined by a cognitive decline that exceeds what is expected for the patient’s age and educational level, yet does not yet meet the diagnostic criteria for dementia [1,2]. Given the high prevalence and the risk of progression toward Alzheimer’s disease, identifying sustainable tools for the monitoring and support of this population is crucial [3,4]. In this context, Extended Reality (XR) technologies have emerged as promising paradigms for delivering cognitive and motor interventions [5,6]. However, the scientific literature often treats ‘Virtual Reality’ as a monolithic construct [7], overlooking how diverse hardware interfaces significantly influence User Experience (UX) and, consequently, the clinical feasibility of the interventions themselves [8]. To effectively guide the selection of clinical platforms, it is essential to distinguish between the varying degrees of immersion along the virtuality continuum, as each presents specific barriers and relative strengths documented in the literature.

Acceptance and usability are essential predictive factors for the long-term sustainability of any technological intervention, particularly in gerontechnology [9]. Technology acceptance is not homogeneous within the elderly population. Interindividual differences in prior experience and digital competence play a crucial role as moderating variables. Digital Literacy (DL), defined as the ability to access, manage, and comprehend digital information, is a determining factor for the adoption of complex interfaces [10,11]. In addition to DL, demographic and experience variables act as covariates and potential moderators of technology acceptance. Variables such as age, education level, and prior history of interaction with VR or touchscreen technology may be critical determinants of acceptance among people with MCI.

Although usability and clinical efficacy are commonly emphasized, the translation of VR/XR interventions into routine clinical practice also depends on practical deployment and sustainability considerations. Implementation studies and scoping reviews indicate that adoption of VR in healthcare is influenced not only by patient engagement, but also by organizational readiness, staff training, technical support, infrastructure, cost, and workflow integration [12,13]. These issues are particularly relevant when comparing different XR device–task configurations, because non-immersive displays, CAVE systems, and HMDs differ substantially in initial layout/setup costs, space requirements, hardware longevity, cleaning and sanitation procedures, software updating, troubleshooting burden, and availability of technical support. Consequently, evaluating technology acceptance without considering real-world sustainability may lead to overly optimistic assumptions about the suitability of specific XR device–task configurations for routine cognitive care [14,15]. Future studies should therefore include implementation-oriented outcomes, such as staff time, maintenance requirements, setup burden, device durability, sanitation procedures, and cost-effectiveness, alongside user acceptability and clinical outcomes.

While the potential of XR is widely recognized, optimizing its clinical application requires a deeper understanding of the existing literature, the results achieved so far, and the open challenges regarding different hardware interfaces.

### 1.1. Current State of XR Technologies in Cognitive Care

To properly contextualize the use of digital interfaces in cognitive care, the current state of the art can be analyzed across three main technological paradigms: non-immersive screens, semi-immersive systems, and fully immersive headsets.

#### 1.1.1. Non-Immersive Interfaces: Touchscreen Displays

At the non-immersive end of the spectrum, Touch TV and tablet systems offer 2D interaction through touchscreen interfaces.

Pilot studies have demonstrated positive acceptance of touchscreen-based neuropsychological batteries among individuals with MCI and dementia [16]. These systems establish a benchmark for high usability, low cognitive load, and a very low barrier to entry for older adults. The primary limitation of non-immersive displays is their low ecological validity. Standard 2D screens fail to replicate the spatial complexity of everyday environments, restricting the types of cognitive exercises to abstract or simplified tasks that do not fully stimulate real-world instrumental activities of daily living (IADLs).

#### 1.1.2. Semi-Immersive Interfaces: CAVE Systems

Progressing toward intermediate levels of immersion, the Cave Automatic Virtual Environment (CAVE) utilizes multi-wall projections and tracking systems to provide a semi-immersive experience.

Studies employing CAVE systems in geriatrics demonstrate that they successfully bridge the gap between immersion and safety [17]. A key benefit of the CAVE is that it allows for a shared virtual environment without physically isolating the user, thereby facilitating the integration of social interactions and direct communication with the therapist [18].

The primary weaknesses associated with CAVE systems are their high cost, substantial space requirements, and complex technical maintenance. Consequently, their accessibility in standard clinical settings remains extremely limited, and research on their specific usability for MCI cohorts is still sparse compared to other devices.

#### 1.1.3. Fully Immersive Interfaces: Head-Mounted Displays (HMDs)

Conversely, the maximum level of virtual presence is achieved through Head-Mounted Displays (HMDs), which completely replace the user’s physical surroundings with a digital environment.

This sensory occlusion can contribute to concerns about patient isolation and reduced awareness of the real environment, potentially increasing disorientation and safety-related anxiety, particularly in vulnerable older adults. Moreover, HMD comfort issues are also reported in the literature, including discomfort related to device weight and fit, pressure points, heat buildup, and difficulties maintaining a stable and comfortable headset positioning over repeated sessions [14,19,20]. These factors may interact with individual susceptibility to cybersickness and with the novelty of the experience, influencing tolerability and leading to higher drop-out rates in clinical protocols [19,20].

#### 1.1.4. The Literature Gap: Limited Within-Cohort Evaluation of XR Delivery Configurations

Despite the proliferation of XR technologies, a critical gap remains in the literature. Previous studies have predominantly focused on the feasibility of single platforms, often comparing HMDs with traditional paper-and-pencil tasks or non-immersive screens, and have usually measured clinical outcomes or basic usability in isolation [21,22].

Studies directly examining different XR delivery interfaces within the same clinical cohort remain limited [23]. As highlighted in the literature [17], the choice of delivery interface can shape the user’s interaction, perceived comfort, and cognitive engagement. However, in real-world clinical development, hardware characteristics, interaction modality, and task content are often tightly linked. Accordingly, the present study was designed as an exploratory within-cohort evaluation of three integrated device–task configurations, Touch TV, CAVE, and HMD, rather than as a definitive hardware-only comparison. The aim was to describe perceived ease of use, and future-use preference in individuals with MCI and cognitively healthy controls.

The present study was not designed to compare XR platforms or to isolate the independent effects of hardware, immersion level, interaction modality, or task content. Its objective was to describe whether individuals with MCI and cognitively healthy controls could complete supervised exposure to three clinically realistic XR device–task configurations, to summarize their immediate subjective acceptability ratings, and to identify methodological constraints that should inform the design of future controlled XR studies in this population.

## 2. Materials and Methods

The present observational study employed a convenience sample of participants, based on their clinical classification (absence of dementia defined as a score of 0 on the Clinical Dementia Rating Scale (CDR), or the presence of MCI, defined as a CDR score of 0.5) [24], recruited from a cohort of people referred to the Neurological Diagnostics Service within the Department of Senile Cerebral Involution at the reference Scientific Institute for Research, Hospitalization, and Healthcare (IRCCS). The study was conducted within an IRCCS clinical-research environment specialized in neurocognitive disorders and characterized by multidisciplinary, patient-centered assessment and rehabilitation pathways. This setting had prior experience with technology-supported clinical activities, which may have facilitated the supervised implementation of the XR device–task configurations and should be considered when interpreting the acceptability findings.

### 2.1. Participants

Neuropsychological assessment was conducted by a licensed neuropsychologist affiliated with the clinical neurocognitive disorders service.

Research-related psychological support and study-related procedures were managed by a research psychologist affiliated with the institution’s clinical research team.

The neuropsychologist conducted a preliminary screening for the application of the study’s inclusion and exclusion criteria. The inclusion criteria were the absence of dementia or the presence of MCI. Exclusion criteria included the presence of photosensitive epilepsy, acute post-stroke phase, convulsive disorders, binocular vision defects (such as strabismus and amblyopia), severe ocular pathologies, and significant vestibular instability (e.g., severe vertigo, labyrinthitis, or inner ear disorders that could exacerbate cybersickness). Additionally, participants were excluded if they had cervical spine issues (including neck muscle weakness, recent trauma, or fractures) or paresis of the upper or lower limbs, as well as a CDR greater than 0.5.

These criteria were implemented to minimize participant safety risks and improve tolerability during immersive XR device–task configuration exposure. In particular, the VR/XR stimulation may include rapidly changing visual scenes and strong multisensory input, which can increase the likelihood of adverse events. Participants were excluded if they had photosensitive epilepsy or other convulsive disorders, given the potential for visually induced seizures. Acute post-stroke phase was excluded to reduce exposure-related risk during a period of heightened clinical instability. Binocular vision defects (e.g., strabismus, amblyopia) and severe ocular pathologies were excluded because visual mismatches and ocular strain can worsen discomfort and disorientation in immersive displays. Significant vestibular instability (e.g., severe vertigo, labyrinthitis, or inner ear disorders) was excluded to prevent exacerbation of symptoms and to reduce the risk of severe cybersickness (e.g., dizziness and nausea). Finally, participants were excluded if they had cervical spine issues (including neck muscle weakness, recent trauma, or fractures) or paresis of upper or lower limbs, because these conditions could compromise safe posture maintenance during headset/CAVE exposure and increase the risk of musculoskeletal strain or inability to complete the procedure consistently.

Subjects who met all inclusion criteria were referred to the research psychologist. This professional provided each potential participant with comprehensive in-formation regarding the project’s objectives, procedures, risks, and benefits. Particular attention was devoted to ascertaining the adequate comprehension of the research protocol and a genuine interest in participating. Following a positive outcome of the comprehension assessment and expression of interest, the participant proceeded to sign the informed consent form, thus formalizing enrollment in the study.

For all potential participants, especially those presenting with MCI, the oral presentation of research-related information was delivered using simple vocabulary, short sentences, and a slow expository pace to allow the individual to assimilate the concepts and elaborate any potential questions. To verify the potential participant’s comprehension, open-ended questions regarding the purpose, procedures, and benefits of the project were formulated, to which the individual was required to respond.

In case of a positive response formulated in their own words, comprehension was confirmed. If responses were incorrect or incomplete, the psychologist corrected them and reformulated the open-ended question related to that topic until the participant provided a correct answer.

Furthermore, the participant was informed that their decision (whether to participate or refuse) would not in any way affect the quality of the diagnostic service they would receive at the IRCCS. A reflection period of 24 h was also granted between the presentation of the information and the actual signing, encouraging the participant to discuss the decision with family members or trusted persons. The signing of the in-formed consent took place only after the reflection period, in the presence of the psychologist.

The sample consisted of people admitted to the Neurological Diagnostics Service of the Department of Senile Cerebral Involution (IRCCS Oasi Maria SS.). Participants were assigned to two groups: people with MCI (*N* = 21) and people with normal intellectual functioning (*N* = 10). The characteristics of the two groups are presented in Table 1.

### 2.2. Instruments

Established models such as the Technology Acceptance Model (TAM) [25] and the Unified Theory of Acceptance and Use of Technology (UTAUT) [26] are widely used frameworks for assessing technology acceptance. However, their full implementation can involve relatively abstract constructs and multiple items, which may increase cognitive burden and fatigue in elderly participants or individuals with early cognitive decline. For this exploratory study, we therefore developed a brief ad hoc instrument, the Digital Immersion Satisfaction Questionnaire (Qu.G.I.D.), to maximize participant compliance and capture simple, clinically relevant aspects of digital background, perceived ease of use, comfort, preference, and willingness to use the tested applications in the future. The instrument is currently undergoing validation; consequently, Qu.G.I.D.-based results should be interpreted as preliminary perceived ease of use measures rather than as psychometrically validated usability outcomes. The instrument is divided into two sections.

#### 2.2.1. Qu.G.I.D. Part 1: Digital Background and Skills

The first section of the questionnaire was designed to detect the level of digital literacy and the participants’ history of interaction with technologies. Its administration took place prior to the main intervention, in order to establish a baseline of digital experience. This first section is composed of seven structured questions designed to collect quantitative and qualitative data related to three macro-areas.

The first part of Qu.G.I.D. was administered once, before exposure to the XR device–task configurations, to assess each participant’s baseline digital profile. It included three domains:

(A) Exposure and Frequency of Device Use: This area investigates the previous and current use of the main digital technologies:
  - Computer/Laptop: usage was recorded dichotomously (YES\NO), along with weekly frequency (every day, 1 day, 2–3 days, or 4–5 days) and average daily usage duration (expressed in hours and minutes).
  - Tablet: similar to the computer, data was collected on usage (YES\NO), weekly frequency (every day, 1 day, 2–3 days, or 4–5 days), and average daily usage duration (expressed in hours and minutes).
  - Mobile Phone/Smartphone: usage was recorded dichotomously (YES\NO), including the specific device type (Non-touchscreen phone vs. Smartphone), weekly frequency (every day, 1 day, 2–3 days, or 4–5 days), and average daily usage duration (expressed in hours and minutes). Qu.G.I.D. assessed a self-reported history of smartphone use (frequency/duration) and did not involve an objective assessment of participants’ smartphone operational ability, as smartphones were not required for study participation.

(B) Functional Digital Skills: This area assesses the participants’ ability to perform practical tasks:
  - TV Programming Ability: ability to use complex remote-control functions (YES\NO).
  - Use of Smart TV and Connected Services: adequate knowledge of advanced television functions (YES\NO, e.g., Apps, streaming services, information).
  - Use of Virtual Consoles: familiarity with interactive gaming consoles (e.g., Nintendo Wii), which require specific motor and cognitive skills in digital interaction (YES\NO).

(C) Formal Training and Learning: A dichotomous variable (YES/NO) was included to detect participation in formal computer training courses.

#### 2.2.2. Qu.G.I.D. Part 2: Post-Interaction Evaluation and Perceived Ease of Use

The second part of Qu.G.I.D. was administered after the experimental exposure to collect post-interaction evaluations of the three tested device–task configurations: Touch TV, CAVE, and HMD. The items were intentionally formulated in simple language and with reduced response options to limit respondent fatigue and facilitate administration in participants with MCI. For the items concerning perceived ease of use, comfort, clarity, irritation, perceived malfunction, drawbacks, and preference, participants were asked to provide separate ratings for each device–task configuration. The first six items assessed perceived satisfaction and perceived ease of use using a 3-point ordinal scale corresponding to “low,” “moderate,” and “high.”

The key variables collected in this section include:

- Overall positive disposition towards the utilization of the exercises;
- The perceived level of comfort and safety during interaction;
- Clarity of system instructions;
- Identification of elements causing user irritation or interaction difficulty;
- Perceived system malfunction or unreliability;
- Identification of specific drawbacks or shortcomings.

Participants were also asked to rate their preference for each device–task configuration using a 4-point ordinal scale, where 1 indicated the least preferred and 4 the most preferred device–task configuration.

Finally, willingness and interest in developing further applications were assessed using a YES/NO response. When participants answered affirmatively, they were asked to indicate which device–task configuration or configurations they considered suitable for future implementation. Because more than one option could be selected, these responses were treated as non-mutually exclusive.

### 2.3. Digital Devices and Applications

The device–task configurations used in this study differed in their levels of immersiveness.

**Figure 1:**
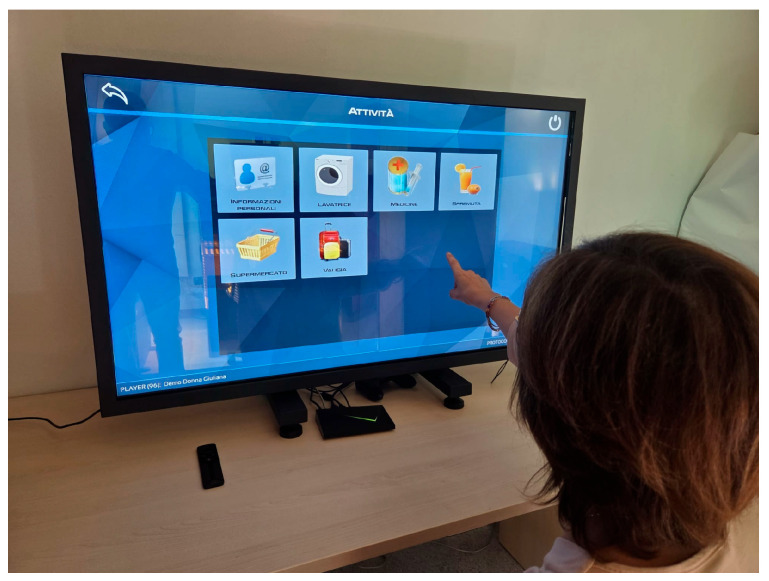
Touch TV system.

The Touch TV system represented the non-immersive configuration. It consisted of a 32-inch television equipped with a TablerTV overlay touch display (Samsung, 32″), transforming the screen into a large multi-touch interface. The system was connected to an NVIDIA SHIELD TV console (NVIDIA Corporation, Santa Clara, CA, USA) running the Android TV operating system [27], which enabled execution of the customized applications designed for direct touch interaction (Figure 1).

**Figure 2:**
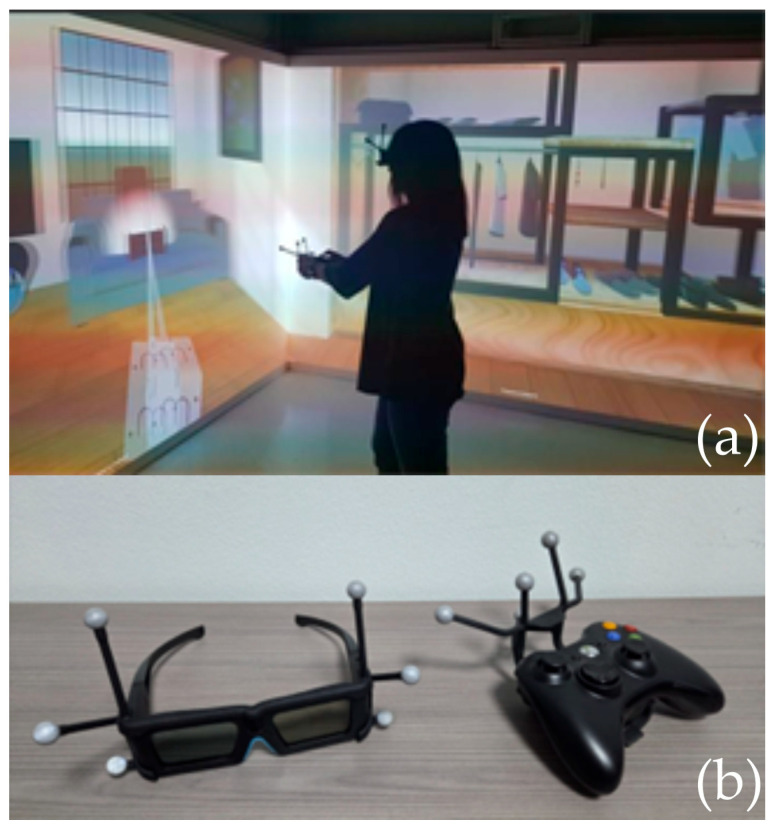
(**a**) The Cave Automatic Virtual Environment (CAVE) system; (**b**) Xbox controller and active stereoscopic glasses used for interaction in the CAVE.

The Cave Automatic Virtual Environment (CAVE) represented the semi-immersive configuration. The system used in the present study was a two-wall stereoscopic projection environment managed by two dedicated Windows-based workstations. The CAVE was a custom-built, two-wall stereoscopic projection system installed at the Institute. Because the system was assembled from multiple hardware components rather than purchased as a single commercial CAVE unit, the relevant projection, tracking, interaction, stereoscopic visualization, and rendering components are specified below. The hardware setup included two projectors (Barco F50; Barco, Kortrijk, Belgium), a four-camera optical motion-tracking system (Trackpack/E, Advanced Realtime Tracking GmbH & Co. KG, Weilheim, Germany), active stereoscopic 3D glasses (Volfoni 3D RF, Volfoni SAS, Paris, France), an Xbox wireless controller (Microsoft Corporation, Redmond, WA, USA), and a 5.1 surround audio system (Logitech Z906 5.1, Logitech, Lausanne, Switzerland). The Xbox controller and the active 3D glasses were equipped with tracking markers detected by the motion-tracking system, allowing interaction with objects in the virtual scene and perspective adaptation to the user’s position (Figure 2).

The virtual environments were developed in Unity3D (Unity 6.3 LTS; Unity Technologies, San Francisco, CA, USA) and rendered in the CAVE through the MiddleVR plugin for Unity (MiddleVR, Paris, France) [28], which enabled multi-display cluster synchronization, stereoscopic rendering, tracking integration, and perspective warping based on the user’s head position.

**Figure 3:**
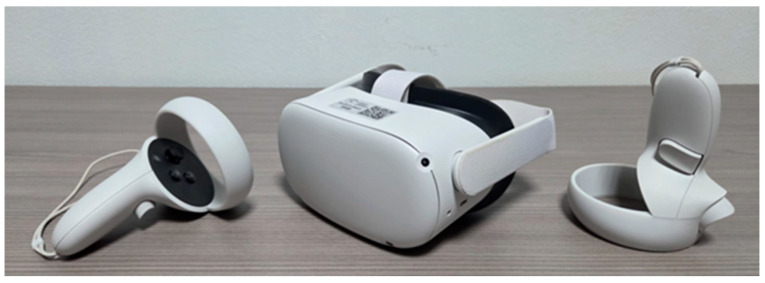
The Meta Quest 2 and its two controllers.

The HMD configuration used a Meta Quest 2 headset (Meta Platforms, Inc., Menlo Park, CA, USA) [29], representing the fully immersive condition. The device provides first-person visualization of a three-dimensional environment and gives users the perception of being inside the displayed scene. It includes integrated cameras for inside-out spatial tracking, built-in motion sensors for head-movement detection, and two handheld controllers for interaction with virtual objects. Head and controller movements were interpreted within the Unity3D environment to synchronize the virtual perspective and interaction events with the user’s movements (Figure 3). The Meta Quest 2 operates on an Android-based system and allows execution of applications developed through Unity3D.

For each device used in this study, specific applications targeting Instrumental Activities of Daily Living (I-ADL) were designed and developed by a software engineer from the Institute. In particular, tasks were selected that were intrinsically linked to the technical features of each platform to assess user experience within the most realistic scenarios. Consequently, the apps Supermarket and Making Orange Juice were implemented on the Touch TV, as this device is ideal for 2D tasks requiring manual precision and hand-eye coordination. The apps Packing a Suitcase and Taking Medication were executed in the CAVE, which is well-suited for spatial orientation tasks and activities of daily living (ADLs) within a controlled environment. Furthermore, the apps Waste Sorting and Setting the Table were implemented on the VR Headset (Meta Quest 2), an optimal device for total immersion and isolation from external stimuli.

This design choice has important interpretative implications. The study did not implement a fully crossed task-by-device design, and the same activities were not administered on all three platforms. Therefore, device type, level of immersion, interaction modality, and task content were not experimentally separable. For this reason, the three experimental conditions are interpreted throughout the manuscript as integrated device–task configurations rather than as isolated hardware platforms. The study was designed to generate preliminary acceptability and self-report acceptability information for these clinically realistic device–task configurations, not to determine the independent effect of hardware type or immersion level.

A brief description of the administered applications is provided below.

#### 2.3.1. App Supermarket

The Supermarket application consists of three interactive scenes.

**Figure 4:**
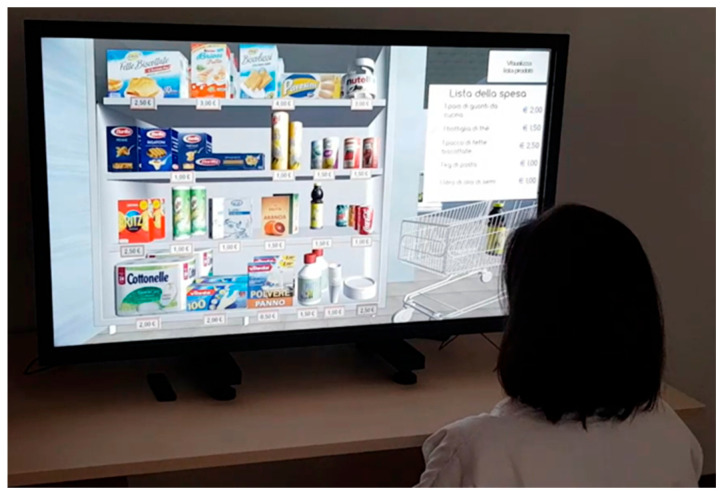
App Supermarket on Touch TV.

1. In the first scene, a domestic kitchen environment is displayed, where the participant must read and then collect a shopping list (comprising five items), the required amount of money, and a wallet.
2. In the second scene, a supermarket aisle is shown with shelves stocked with various products, including those on the shopping list. The participant is required to drag the correct items from the shelves into a shopping cart (Figure 4).
3. In the final scene, the participant must place the purchased items from the cart onto the checkout conveyor belt and pay for them by selecting and handing over the correct banknotes from the wallet to the cashier.

#### 2.3.2. App Making a Juice

**Figure 5:**
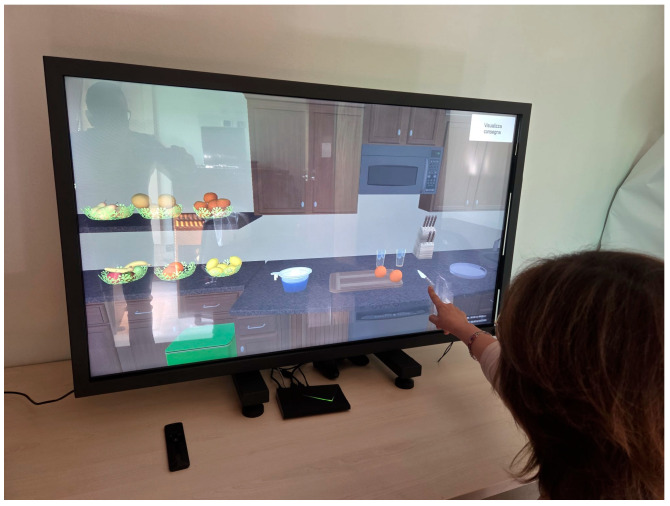
App Making a Juice on Touch TV.

In this activity, the participant is asked to prepare an orange juice for two people using a combination of two citrus fruits randomly indicated at the beginning of the task. The scene depicts a kitchen with a table, cupboards, sink, and various utensils. The participant must complete all steps necessary for preparing the juice, including selecting the correct fruits, using the appropriate tools, pouring the juice into glasses, and cleaning the workspace afterwards (Figure 5).

#### 2.3.3. App Packing a Suitcase

**Figure 6:**
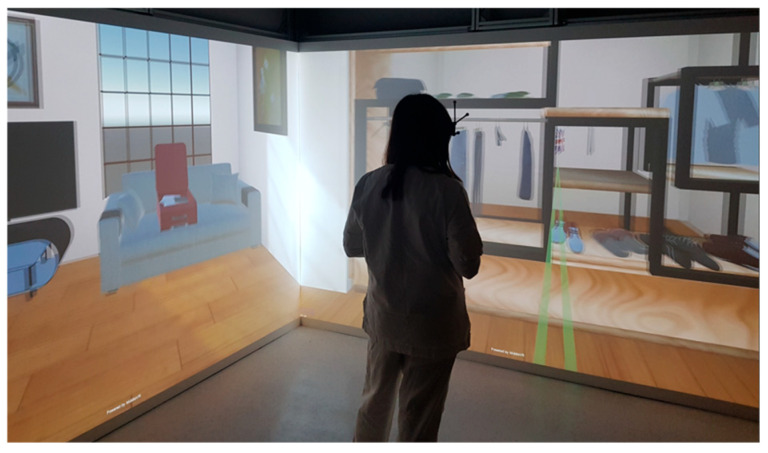
App Packing a Suitcase on CAVE.

The Packing a Suitcase application requires the participant to prepare a suitcase for a weekend trip. The scene depicts a room with shelves containing different clothing items, which must be dragged and placed into the suitcase. Depending on the participant’s profile, the scene may include either male or female clothing (Figure 6).

#### 2.3.4. App Taking Medications

**Figure 7:**
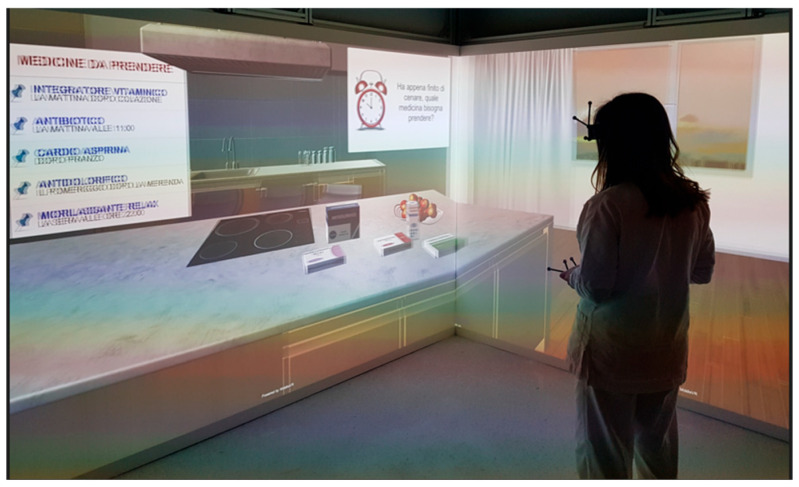
App Taking Medications on CAVE.

In this application, the participant must select the correct medications to take according to the time of day presented. The scene depicts a kitchen table with five boxes of medicine. Verbal and visual instructions, randomly presented, indicate which medication should be taken at a given time. The participant must then touch the box corresponding to the correct medication (Figure 7).

#### 2.3.5. App Waste Sorting

**Figure 8:**
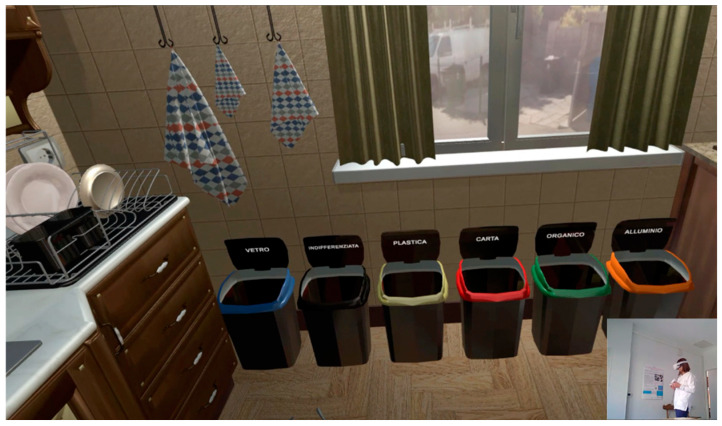
App Waste Sorting on VR Headset.

This 3D application represents a household kitchen with a table and several waste bins labeled for different types of recycling (e.g., organic waste, cardboard, non-recyclable waste). Three waste items of varying types (e.g., tin cans, paper boxes, food scraps) appear on the kitchen table at a time, and the participant must dispose of each item in the appropriate bin (Figure 8).

#### 2.3.6. App Setting the Table

**Figure 9:**
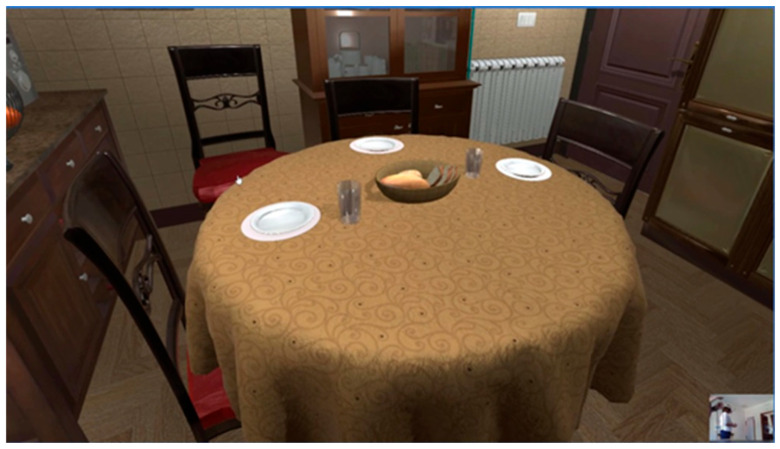
App Setting the Table on VR Headset.

In this application, the participant is placed in a kitchen environment with a table and various utensils located either on the countertop or inside cupboards. The goal of the task is to set the table for a specific number of people, randomly indicated at the start, by performing the correct sequence of actions required for proper table setting (Figure 9).

All applications developed for this study follow the same execution logic, consisting of four main components: initial instruction, demonstration video, reinforcement, and corrective feedback.

The initial instruction is presented both verbally and visually and may be accompanied by an optional demonstration video illustrating the correct sequence of actions required to complete the proposed task.

During task execution, verbal reinforcement (e.g., “well done,” “congratulations,” “keep it up”) is randomly provided following correct responses, serving to enhance motivation and engagement.

In all applications, in the case of incorrect responses, the system delivers visual and verbal corrective feedback suggesting the appropriate action to perform. The feedback becomes progressively more explicit and detailed as the number of repeated errors in the same task increases, thus supporting error correction and learning through adaptive guidance.

Specifically:

1. The first level of assistance consists of a prompt expressed simultaneously through both verbal and written messages (e.g., in the “making a juice” activity, the participant will hear and see the written message “cut an orange”; in the “packing a suitcase” activity, the participant will hear and see the written message “take another piece of clothing”; in the “setting the table” activity, the participant will hear and see the written message “take a glass and place it on the table”).
2. The second level of assistance, in addition to hearing the verbal message and seeing the written message, involves visually highlighting the objects in the scene involved in the action for a few seconds.
3. The third level of assistance, in addition to hearing the audio message and seeing the written message, involves visually highlighting the objects in the scene involved in the action permanently.
4. The fourth and final level of assistance consists of the correct action being automatically performed by the system without any input from the user.

Although the applications included internal event monitoring to trigger adaptive support levels, these logs were not originally standardized or validated as comparative performance endpoints across the three device–task configurations. Therefore, the present exploratory analysis was restricted to the pre-specified subjective acceptability outcomes, including perceived ease of use, comfort, preference, and willingness to use the device–task configurations in future applications. Future studies should prospectively define and harmonize objective metrics, such as completion time, error rate, number and level of prompts, task abandonment, and interaction quality, before using them for comparisons across XR platforms.

### 2.4. Procedure

After obtaining written informed consent, each participant underwent a standardized experimental protocol conducted in a quiet room at the IRCCS by a trained research psychologist. To guarantee strict consistency and control for order or learning effects, the entire session was structured into five sequential phases as follows:

- Phase 1: Baseline Assessment (Approx. 10 min). Participants completed the first section of the Digital Immersion Satisfaction Questionnaire (Qu.G.I.D.) to establish their profile of digital literacy and previous technological exposure.
- Phase 2: Briefing and Familiarization (Approx. 5 min). The psychologist presented a standardized verbal description of the three device–task configurations (Touch TV, CAVE, and HMD) using simplified language tailored to the participant’s cognitive profile. No physical practice was allowed during this phase to avoid pre-exposure bias.
- Phase 3: Randomized Experimental Testing (Approx. 13–15 min total). Participants interacted with all three digital devices and their corresponding I-ADL applications. The presentation sequence of the three device–task configurations was randomized for each subject using a computer-generated random number sequence.
- Touch TV Condition (5 min): Execution of the Supermarket and Making Orange Juice applications.
- CAVE Condition (5 min): Execution of the Packing a Suitcase and Taking Medication applications.
- VR Headset Condition (3 min): Execution of the Waste Sorting and Setting the Table applications.
- Phase 4: Standardized Execution Logic (Within-Task). For every single device–task configurations, the interaction followed a rigid four-step automation architecture to provide adaptive guidance: (1) initial visual/verbal instruction, (2) optional demonstration video, (3) randomized positive reinforcement upon correct actions, and (4) a 4-level progressive assistance algorithm triggered automatically in case of persistent errors.
- Phase 5: Post-Trial Evaluation (Approx. 5–10 min). Immediately following the final experimental interaction, the psychologist administered the second section of the Qu.G.I.D. questionnaire via a structured interview. Participants provided separate ratings for each device–task configuration regarding perceived ease of use, comfort and safety, clarity of instructions, irritation or interaction difficulty, perceived malfunction, drawbacks, and preference. They were also asked whether they would be interested in future applications and, when applicable, which device–task configuration or configurations they considered suitable for future implementation.

### 2.5. Data Analysis

Data were analyzed using non-parametric methods because of the small sample size, the ordinal nature of the questionnaire variables, and the non-normal distribution of several outcomes. Descriptive statistics are reported as mean ± standard deviation and median with interquartile range [IQR], as appropriate. Between-group comparisons between participants with MCI and cognitively healthy controls were performed using the Mann–Whitney U test for continuous or ordinal variables and Fisher’s exact test or χ^2^ test for categorical variables.

Because each participant experienced all three device–task configurations, device-related perceived ease of use and preference scores were analyzed as repeated-measures outcomes. Within-subject comparisons across Touch TV, CAVE, and HMD were performed using the Friedman test, with Kendall’s W reported as an effect-size estimate. When appropriate, pairwise post hoc comparisons were conducted using Wilcoxon signed-rank tests with Holm correction for multiple comparisons.

The perceived ease of use score was recomputed directly from the six item-level responses of the Qu.G.I.D. questionnaire. Since each item was scored from 0 to 2, the total perceived ease of use score ranged from 0 to 12. Additional exploratory analyses were performed on positive perceived ease of use items and on items reflecting discomfort, irritation, malfunction, or drawbacks, in order to better characterize the dimensions contributing to the overall user experience.

Future implementation modality was analyzed separately from preference scores. Since participants could endorse more than one device as suitable for future applications, these binary selections were treated as non-mutually exclusive repeated responses. Therefore, Cochran’s Q test was used to compare the frequency of future selection across the three device–task configurations, followed, when appropriate, by McNemar pairwise comparisons with Holm correction.

Finally, exploratory analyses assessed whether digital literacy was associated with perceived ease of use, preference, or future device selection. Spearman’s rank correlations were used for ordinal outcomes, and univariable logistic regression models were used for binary future-selection outcomes. Given the exploratory nature of the study and the limited sample size, these analyses were interpreted cautiously and were not considered confirmatory.

Statistical significance was set at *p* < 0.05. Analyses were performed using non-parametric and logistic regression procedures appropriate for small-sample exploratory data.

Because of the small and unbalanced sample and the non-counterbalanced task–device structure, all inferential analyses were considered exploratory and descriptive. Statistical tests were used to summarize response patterns within this pilot dataset and should not be interpreted as confirmatory tests of hardware superiority, immersion effects, or comparative clinical effectiveness.

## 3. Results

The study included 31 participants: 21 individuals with MCI and 10 cognitively healthy controls. The two groups did not differ significantly in age, education, sex distribution, or digital literacy. However, because the control group was smaller than the MCI group, these baseline comparisons should be interpreted as descriptive checks rather than evidence of full group equivalence. The MCI group had a mean age of 64.1 ± 11.9 years, whereas the control group had a mean age of 66.0 ± 7.2 years. Median education was 8 years in both groups. Digital literacy scores were also comparable between groups, with a median score of 3.0 [2.0–4.0] in the MCI group and 2.5 [1.0–3.8] in controls.

### 3.1. Perceived Ease of Use Across the Three Device–Task Configurations

Perceived ease of use scores were generally high across the three device–task configurations, with median values close to the upper end of the scale in both groups (Table 2). No significant between-group differences were observed for Touch TV, CAVE, or HMD.

Because the three device–task configurations differed simultaneously in device type, immersion level, interaction modality, task content, and exposure duration, the repeated-measures analyses are reported only as descriptive summaries of response patterns within this pilot sample and should not be interpreted as comparative tests of XR platform perceived ease of use. Exploratory item-level analyses suggested that the HMD condition showed greater variability and a numerically worse response pattern than Touch TV and CAVE, particularly for items related to discomfort, irritation, malfunction, or drawbacks. This pattern was most evident in the MCI group. However, when perceived ease of use was analyzed as a corrected composite score accounting for positive and negatively worded items, the overall configuration effect remained non-significant in the total sample: Friedman χ^2^ = 1.91, *p* = 0.384, Kendall’s *W* = 0.03.

### 3.2. Preference Scores

Preference scores showed a numerical preference for the CAVE device–task configuration in the total sample and in the MCI group (Table 2). These preference data are presented only descriptively, because the design does not allow preference differences to be attributed to the device, immersion level, interaction modality, or task content. In the MCI group, the same pattern was more evident but remained at trend level, whereas no significant device-related preference pattern was observed in the control group (Table 2). Exploratory pairwise comparisons suggested higher preference for CAVE than for HMD in participants with MCI, with a borderline result after Holm correction, *p* = 0.050.

### 3.3. Future Implementation Preference

When participants were asked which devices they would consider suitable for future applications, CAVE was the most frequently selected device–task configuration in both the total sample and the two study groups (Table 3). However, because future modality selections were non-mutually exclusive, the appropriate repeated binary comparison did not show a statistically significant difference among the three device–task configurations: Cochran’s *Q* = 4.11, *p* = 0.128. The same non-significant pattern was observed in the MCI group, *Q* = 2.46, *p* = 0.292, and in controls, *Q* = 2.33, *p* = 0.311. Pairwise McNemar comparisons with Holm correction did not identify significant differences between individual device–task configuration.

### 3.4. Digital Literacy

Exploratory analyses did not provide evidence supporting a positive association between digital literacy and preference for the Touch TV device–task configuration. Specifically, digital literacy was not significantly related to preference scores for Touch TV (Spearman *ρ* = −0.31, *p* = 0.086), nor to preference for the CAVE-based device–task configuration (*ρ* = 0.08, *p* = 0.687) or the HMD (*ρ* = 0.26, *p* = 0.157). Similarly, exploratory univariable logistic regression analyses did not yield statistically robust evidence for an association between digital literacy and the choice of any device–task configuration for future applications (Table 3). Given the exploratory design and the limited sample size, these numerical results should be interpreted cautiously and are not intended to support conclusions regarding relative device–task configuration effectiveness.

### 3.5. Summary of Findings

Overall, the results indicate that all three device–task configurations were feasible to administer and were generally well accepted by participants with MCI and by cognitively healthy controls. No robust between-group differences emerged perceived ease of use ratings, suggesting that, within the limits of this exploratory sample, individuals with MCI were able to tolerate and evaluate the tested device–task configurations. As summarized in Table 2, CAVE showed numerically higher preference scores, especially in the MCI group; however, repeated-measures analyses did not provide statistically robust evidence of superiority over Touch TV or HMD. Because task content and device type were confounded, this pattern should be interpreted as a descriptive signal related to the CAVE-based device–task configuration, not as evidence that the CAVE hardware or semi-immersive level itself was preferred.

## 4. Discussion

The present exploratory pilot study described perceived ease of use, comfort, preference, and future-use selection for three integrated XR device–task configurations in individuals with MCI and cognitively healthy controls. The findings support the self-report acceptability of all three device–task configurations in individuals with MCI. However, because each platform was paired with different activities, the results cannot be used to isolate the effects of hardware type, immersion level, interaction modality, or task content. Therefore, the numerical tendency toward greater CAVE acceptability should be interpreted only as a descriptive signal for the tested CAVE-based device–task configuration, not as evidence of CAVE superiority.

To avoid overinterpretation of the exploratory findings, Table 4 summarizes the interpretative boundaries of the present pilot study, distinguishing what the data can reasonably support from what they cannot establish.

Regarding our primary outcome, the results showed no statistically significant differences between the MCI group and the control group in perceived ease of use (Qu.G.I.D. scores) for any of the three device–task configurations. This suggests that the presence of mild cognitive impairment does not necessarily hinder the ability to interact with or evaluate diverse technological interfaces, ranging from 2D touchscreens to immersive 3D environments.

The homogeneity in perceived ease of use ratings, further supported by similar levels of digital literacy and device usage time between the two groups, indicates that the tested “device–task configurations” were sufficiently intuitive for this clinical population. These findings align with recent perspectives suggesting that well-designed XR interfaces can mask mild cognitive deficits by providing error-tolerant interaction environments [30,31].

While perceived ease of use scores were broadly comparable across groups and device–task configurations, a descriptive pattern emerged in the secondary outcomes. The CAVE device–task configuration was the most frequently selected platform for future applications and showed a numerical preference pattern, particularly among participants with MCI. However, repeated-measures analyses did not demonstrate a statistically robust superiority of CAVE over Touch TV or HMD. Therefore, this finding should be interpreted as a preliminary signal of acceptability rather than as evidence of a definitive platform preference.

The numerical tendency toward CAVE selection should not be mechanistically interpreted. Because the CAVE condition differed from the other device–task configurations not only in immersion level, but also in task content, interaction modality, physical setting, and exposure duration, this pattern can only be regarded as a descriptive observation from the tested CAVE-based device–task configurations. It may be useful for designing future studies, but it does not indicate that semi-immersive systems are intrinsically more acceptable than non-immersive or fully immersive systems in individuals with MCI.

Exploratory analyses were conducted to assess whether higher digital literacy might be associated with preference for the Touch TV device–task configuration. The results did not provide support for the proposed hypothesis: digital literacy was not significantly related to preference scores for Touch TV, CAVE, or HMD.

For the purpose of informing future device selection, the exploratory logistic regression suggested a possible negative trend between digital literacy and Touch TV selection; however, the evidence was weak and should not be interpreted as conclusive. Given the small sample size and the exploratory nature of the analyses, this outcome warrants caution and is not intended to establish systematic differences across configurations. Overall, these exploratory results are consistent with the notion that higher digital literacy does not straightforwardly translate into greater preference for familiar two-dimensional interfaces, but further confirmatory work is needed.

This does not diminish the relevance of familiar touchscreen-based interfaces, which remain practical, accessible, and potentially useful for cognitive and functional applications in older adults and people with MCI or dementia [32,33,34,35,36,37]. Rather, the present findings suggest that platform selection should account for inter-individual heterogeneity in prior technological experience and cognitive interaction habits, instead of assuming a one-size-fits-all relationship between digital competence and preference for a specific interface [35,36,38].

We acknowledge that participants’ positive adoption may have been influenced by contextual and cultural factors associated with the IRCCS clinical-research setting, including participants’ trust in the institution, the supervised nature of the sessions, and local familiarity with technology-supported rehabilitation. We did not measure cultural attitudes or technology-related beliefs; therefore, generalization to other cultural contexts should be made cautiously and supported by replication.

The interpretation of these findings is subject to several major methodological constraints. First, the principal limitation is the complete confounding between device type and task content. The Touch TV, CAVE, and HMD configurations were each paired with different IADL activities, interaction demands, and sensory-motor requirements. Consequently, participants’ perceived ease of use ratings and preferences cannot be attributed specifically to the hardware platform, level of immersion, interaction modality, or task content. The findings therefore concern the acceptability of the tested device–task configurations and do not provide evidence for the independent superiority of any XR platform.

Second, the study used a small, unbalanced convenience sample and was not supported by an a priori power analysis. The sample size may have limited the ability to detect subtle group or configuration effects, increased the risk of type II error, and reduced the reliability of exploratory moderation analyses. The findings should therefore be considered hypothesis-generating and not generalizable to the broader MCI population without replication. In addition to the overall small convenience sample, the uneven group sizes (21 MCI vs. 10 controls) may have further limited the ability to detect subtle between-group differences and secondary effects. While baseline variables were comparable, this imbalance reduces the robustness of statistical comparisons and increases the likelihood that non-significant results reflect limited power rather than true equivalence.

Third, the main perceived ease of use instrument was an ad hoc questionnaire that has not yet undergone full external psychometric validation. Although Qu.G.I.D. was intentionally designed as a brief and accessible tool to reduce cognitive burden in participants with MCI, it does not yet provide the cross-study comparability of validated instruments such as the System Usability Scale [39] or established technology-acceptance measures. In addition, the use of 3-point and 4-point response scales may have reduced sensitivity and contributed to ceiling effects.

Fourth, the study relied almost entirely on subjective ratings. Objective endpoints such as completion time, error rate, number and level of prompts, task abandonment, tracking-derived interaction quality, cybersickness, physiological stress proxies, and cognitive load were not analyzed as standardized comparative outcomes [40]. Therefore, the present exploratory analysis was restricted to the pre-specified subjective acceptability outcomes, including perceived usability, comfort, preference, and willingness to use the configurations in future applications. Future studies should prospectively define and harmonize objective metrics, such as completion time, error rate, number and level of prompts, task abandonment, and interaction quality, before using them for comparisons across XR platforms.

A definitive comparative study should therefore use a larger and balanced sample, validated usability and technology-acceptance instruments, systematic cybersickness assessment, and prospectively defined objective interaction metrics [41]. Most importantly, future studies should use a fully counterbalanced or partially counterbalanced task-by-device design in which identical or closely matched activities are implemented across Touch TV, CAVE, and HMD. Only such a design would allow separation of hardware effects from task-content effects and support reliable conclusions about the comparative usability of XR platforms in MCI.

## 5. Conclusions

In summary, this exploratory pilot study provides preliminary descriptive evidence that three XR device–task configurations, Touch TV, CAVE, and HMD, can be administered under supervised conditions and are generally acceptable to individuals with MCI. The CAVE-based device–task configuration was numerically the most frequently selected for future applications and showed a descriptive tendency toward higher preference in the MCI group, but corrected repeated-measures analyses did not demonstrate statistically robust superiority over the other device–task configurations. Because device type, level of immersion, interaction modality, and task content were not experimentally separable, these findings should not be interpreted as evidence of comparative platform effectiveness or as support for hypotheses for future controlled studies regarding one XR platform over another. Rather, the study should be viewed as a self-report acceptability that may inform the design of future trials. Larger, balanced, counterbalanced studies using validated psychometric instruments, objective performance and interaction-quality metrics, and systematic cybersickness assessment are required before reliable conclusions can be drawn about comparative XR usability in MCI.

## Figures and Tables

**Table 1:** Demographic and clinical characteristics of participants with and without MCI.

	People with MCI CDR 0.5			People with Normal Intellectual Functioning CDR 0		
Variables	*N* (%)	Mean ± SD	Range	*N* (%)	Mean ± SD	Range
Sample	21			10		
Age		64.1 ± 11.9	41–86		66 ± 7.2	56–76
Sex						
Male	13 (61.90)			3 (30)		
Female	8 (38.10)			7 (70)		
Educational level						
Primary school (0–5)	6 (28.57)			4 (40)		
Middle school (6–8)	7 (33.33)			2 (20)		
High school (9–13)	4 (19.05)			3 (30)		
University (>13)	4 (19.05)			1 (10)		
Profession						
Homemaker	7 (33.33)			4 (40)		
Freelancer	2 (9.52)			0 (0)		
Craftsman	3 (14.29)			1 (10)		
Technician	1 (4.77)			1 (10)		
Clerk	6 (28.57)			4 (40)		
Director	0 (0)			0 (0)		
Worker	2 (9.52)			0 (0)		

Note: Educational level is expressed as years of formal schooling: 0–5 = primary school; 6–8 = lower secondary/middle school; 9–13 = upper secondary/high school; >13 = university or higher education. MCI = mild cognitive impairment; CDR = Clinical Dementia Rating; SD = standard deviation. Profession categories are self-reported broad occupational categories. “Homemaker” refers to individuals mainly engaged in domestic/family care activities; “freelancer” to self-employed professionals; “craftsman” to skilled manual/artisanal work; “technician” to technical or specialized applied work; “clerk” to administrative/office work; “director” to managerial roles; and “worker” to non-managerial manual or industrial work.

**Table 2:** Perceived ease of use and preference scores across the three technological configurations.

Outcome	Sample	Touch TV Median [IQR]	CAVE Median [IQR]	HMD Median [IQR]	Friedman χ^2^	*p*	Kendall’s *W*
Perceived ease of use score	Total sample	11.0 [10.0–12.0]	11.0 [9.0–12.0]	10.0 [8.0–12.0]	3.35	0.187	0.05
	MCI	11.0 [10.0–12.0]	11.0 [9.0–12.0]	10.0 [8.0–11.0]	5.48	0.065	0.13
	Controls	11.5 [10.0–12.0]	11.0 [10.3–12.0]	11.5 [10.0–12.0]	1.2	0.549	0.06
Preference score	Total sample	2.0 [1.0–2.5]	2.0 [2.0–3.0]	1.0 [1.0–3.0]	4.44	0.109	0.07
	MCI	2.0 [1.0–3.0]	2.0 [2.0–3.0]	1.0 [1.0–3.0]	5.43	0.066	0.13
	Controls	1.5 [1.0–2.0]	2.0 [2.0–2.8]	3.0 [1.0–3.0]	1.38	0.5	0.07

Note: Values are reported as median [interquartile range]. Perceived ease of use scores range from 0 to 12; preference scores range from 1 to 4. HMD = head-mounted display; MCI = mild cognitive impairment; IQR = interquartile range.

**Table 3:** Future implementation selection and exploratory associations with digital literacy.

	For Future Applications						
Device	MCI n (%)	Controls n (%)	Spearman ρ with Digital Literacy	*p*	Logistic OR for Future Selection	95% CI	*p*
Touch TV	11 (52.4%)	5 (50.0%)	−0.38	0.035	0.6	0.35–1.01	0.056
CAVE	15/(71.4%)	8 (80.0%)	0	1	1	0.63–1.59	0.994
HMD	11 (52.4%)	7 (70.0%)	0.09	0.617	1.18	0.77–1.83	0.446

Note: Future implementation selections were non-mutually exclusive; therefore, differences across devices were analyzed using Cochran’s *Q*. Total sample: *Q* = 4.11, *p* = 0.128; MCI group: *Q* = 2.46, *p* = 0.292; controls: *Q* = 2.33, *p* = 0.311. Logistic ORs refer to the change in odds of selecting each device for future applications per one-point increase in digital literacy. HMD = head-mounted display; MCI = mild cognitive impairment; CI = confidence interval.

**Table 4:** Interpretative boundaries of the present exploratory pilot study.

What the Study Can Support	What the Study Cannot Support
Short supervised exposure to three XR device–task configurations was feasible in this clinical-research setting.	Comparative superiority of Touch TV, CAVE, or HMD.
Participants with MCI were able to complete the procedure and provide simple subjective acceptability ratings.	Independent effects of device type, immersion level, interaction modality, task content, or exposure duration.
The tested configurations were generally acceptable under supervised conditions.	Psychometrically validated conclusions about usability, because Qu.G.I.D. has not yet undergone full external validation.
The findings may inform the design of future XR protocols in MCI.	Conclusions about clinical efficacy, objective interaction performance, cybersickness burden, cognitive load, or implementation effectiveness.
The study identifies methodological requirements for future controlled research.	Hypotheses about platform-specific mechanisms or real-world effectiveness without a counterbalanced design and standardized outcomes.

## Data Availability

The data presented in this study are available in https://data.mendeley.com/datasets/dky3xz9p6w/1 (accessed on 28 October 2025).
